# Renal artery covered stent placement eliminated false lumen perfusion after provisional extension to induce complete attachment

**DOI:** 10.1016/j.jvscit.2026.102503

**Published:** 2026-09-06

**Authors:** Keiichi Ishida, Yoshiaki Katada, Hitoshi Nakanowatari, Ikuko Shibasaki, Yoshihito Irie

**Affiliations:** a Department of Cardiovascular Surgery, Iwaki City Medical Center, Iwaki, Japan; b Department of Radiology, Tokyo Medical University Ibaraki Medical Center, Ibaraki, Japan

**Keywords:** Renal artery covered stent placement, PETTICOAT, Thoracic endovascular aortic repair, Aortic dissection

## Abstract

Thoracic endovascular aortic repair with the provisional extension to induce complete attachment (PETTICOAT) technique can cause persistent false-lumen perfusion through distal re-entry tears, leading to late aneurysmal degeneration and challenging reinterventions. We report the use of renal artery covered stent placement to treat persistent false-lumen perfusion after PETTICOAT caused by a re-entry tear in a 57-year-old man with chronic type B aortic dissection. Preoperative assessment of the relationship between the re-entry tear and bare-stent struts enabled device delivery and false-lumen exclusion, while preserving renal perfusion. This approach represents a feasible branch-preserving reintervention strategy after PETTICOAT.

Thoracic endovascular aortic repair (TEVAR) with distal bare-metal stents, known as the provisional extension to induce complete attachment (PETTICOAT) technique, promotes aortic remodeling and improves malperfusion in complicated type B aortic dissection. However, persistent false-lumen perfusion through distal re-entry tears may cause late aneurysmal degeneration. Secondary endovascular intervention after PETTICOAT repair is technically challenging because of the pre-existing bare-metal stent. We report a case where renal artery covered stent placement successfully treated persistent false-lumen perfusion caused by a post-PETTICOAT renal artery re-entry tear. Patient consent was obtained for publication, and there is potentially identifiable information in this study.

## Case report

A 57-year-old man underwent translocated total arch replacement with frozen elephant trunk reconstruction, with distal anastomosis in zone 2, for a dissecting thoracic aortic aneurysm 135 months previously. He had chronic type B aortic dissection with a patent false lumen and a primary entry tear at the left subclavian artery, with aneurysmal degeneration from the distal arch to the descending thoracic aorta. The false-lumen distal to the midabdominal aorta was thrombosed, whereas the true lumen was diffusely narrowed, with severe stenosis at the left common iliac artery origin. Postoperative computed tomography (CT) confirmed exclusion of the proximal entry tear and false-lumen thrombosis from the distal arch to the mid-descending thoracic aorta. However, residual false-lumen perfusion persisted through re-entry tears at the lower-intercostal arteries and a punched-out lesion of the left renal artery. Progressive enlargement of the descending dissecting aneurysm subsequently occurred. Nine months after initial surgery, TEVAR was performed using 36 × 150-mm (Relay; Terumo Aortic) and 34 × 197-mm (Zenith TX2; Cook Medical) stent grafts extending from the frozen elephant trunk to just above the celiac artery. CT demonstrated false-lumen thrombosis and aneurysm shrinkage, but severe true-lumen narrowing persisted. Seven months later, PETTICOAT TEVAR was performed using a 46 × 164-mm bare-metal dissection stent (Zenith TXD; Cook Medical), extending from the previous graft to the terminal aorta, resulting in expansion of the left common iliac artery true lumen. Two years after PETTICOAT repair, follow-up CT demonstrated enlargement of the descending dissecting aneurysm to 61.5 mm because of persistent false-lumen perfusion through a punched-out re-entry tear adjacent to the left renal artery within the PETTICOAT bare-metal stent segment (Fig 1). Although a thoracoabdominal aortic replacement was considered, the patient strongly preferred an endovascular approach. Renal artery covered stent placement for left renal artery re-entry was planned to achieve false-lumen exclusion while preserving renal perfusion.

**Fig 1:**
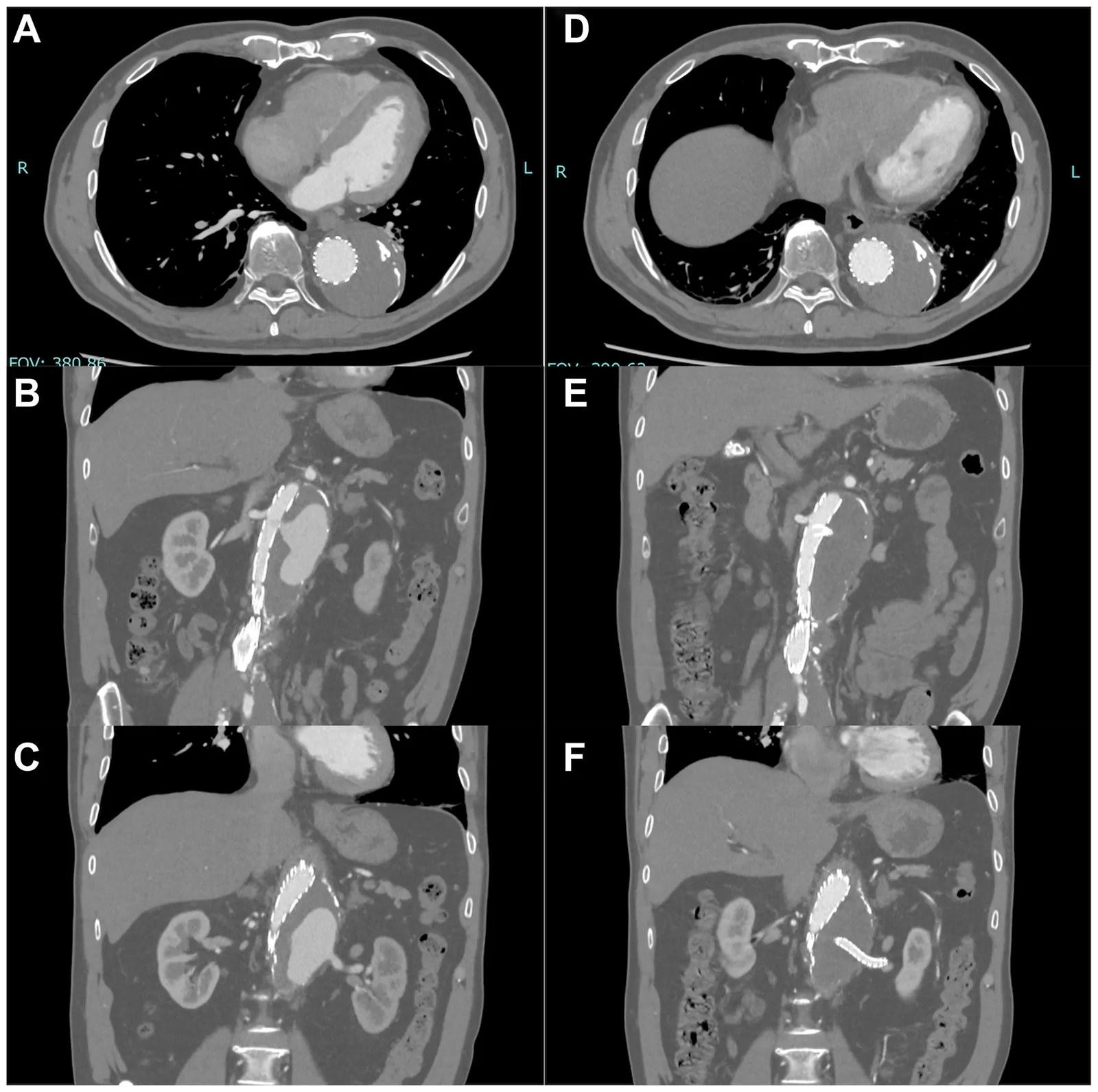
Preoperative computed tomography (CT) identified persistent false-lumen perfusion through a left renal artery re-entry tear covered by the provisional extension to induce complete attachment (PETTICOAT) bare-metal stent **(A-C)**. Postoperative CT confirmed false-lumen thrombosis and preserved renal perfusion **(D-F)**.

Under general anesthesia, bilateral femoral access was established using 5F sheaths. A 5F diagnostic catheter (Cobra type; Medikit Co, Ltd) was advanced through the punched lesion into a false lumen. Angiography revealed a large residual false lumen connected to the left renal artery without visualization of the other branch vessels (Fig 2). The left sheath was replaced with a 7F 45-cm guiding sheath (Destination Guiding Sheath; Terumo Corporation). A 0.035-inch guidewire (Radifocus; Terumo Corporation) was advanced deep into the false lumen, followed by careful advancement of the guiding sheath using a dilator. The left renal artery was selected using a 5F diagnostic catheter (Cook Medical) and a 0.035-inch guidewire. After catheter advancement, the guidewire was replaced with a 0.035-inch guidewire (Rosen; Cook Medical). A 7- × 79-mm balloon-expandable stent graft (VBX; W. L. Gore & Associates) was inserted at nominal pressure, with one stent ring intentionally introduced into the true lumen. During deployment, straightening of the renal artery landing zone caused a slight proximal migration of the VBX toward the true lumen. This shift left two stent rings protruding into the true lumen, but did not limit flow. The protruding segment was flared using a 10-mm balloon catheter.

**Fig 2:**
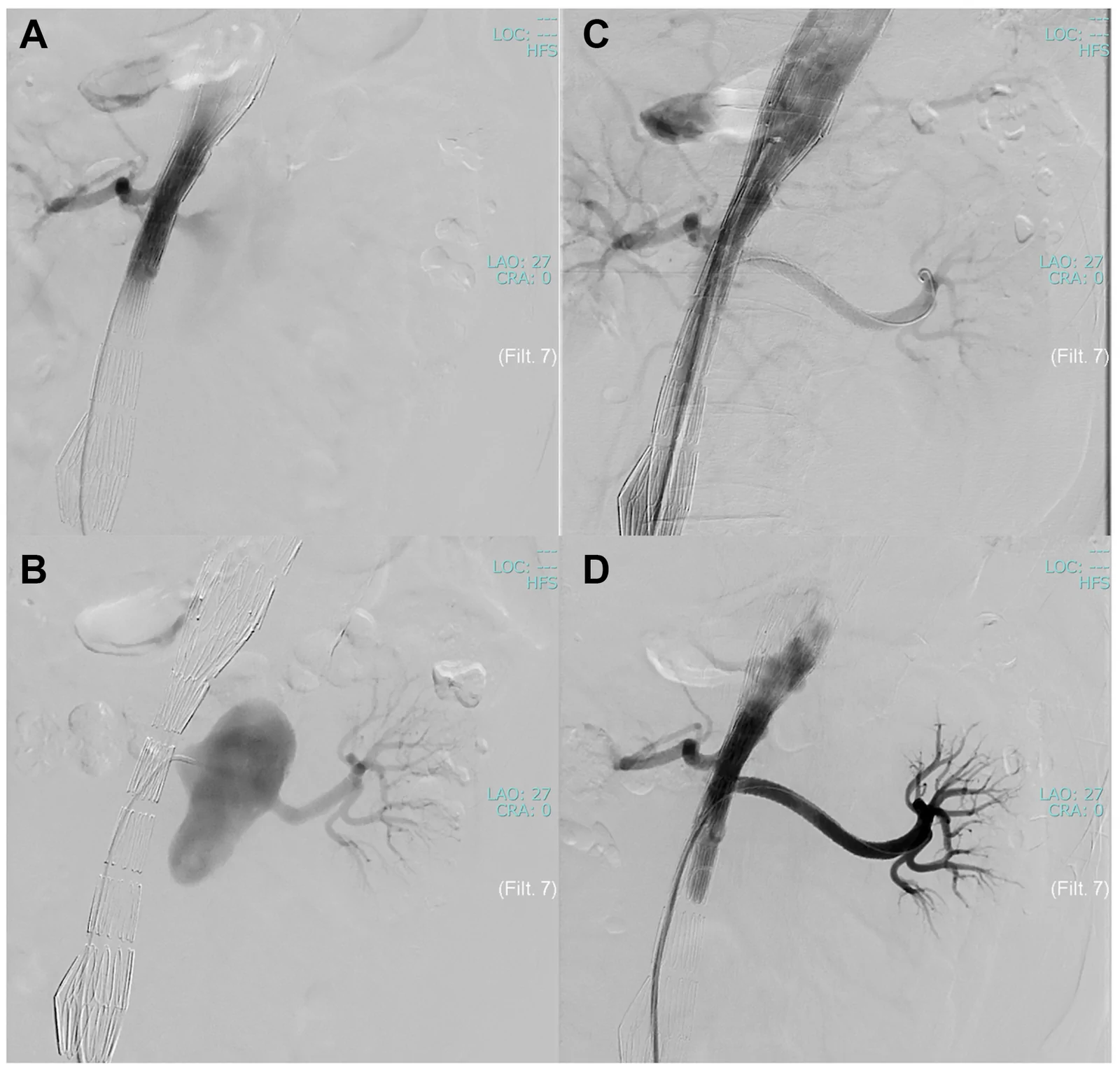
Predeployment angiography revealed a large residual false lumen connected to the left renal artery **(A** and **B)**. Postdeployment angiography confirmed complete elimination of false-lumen perfusion without residual flow around the left renal artery landing zone **(C** and **D)**.

Final angiography demonstrated the complete elimination of false-lumen perfusion. Postoperative CT showed no false-lumen enhancement and preserved renal perfusion. The maximum aneurysm diameter decreased from 61.5 mm preoperatively to 60.4 mm at 3 months and remained stable at 6 months (Fig 1). Three-dimensional CT confirmed that the VBX traversed the re-entry tear near the base of a downward-facing PETTICOAT stent cell (Fig 3).

**Fig 3:**
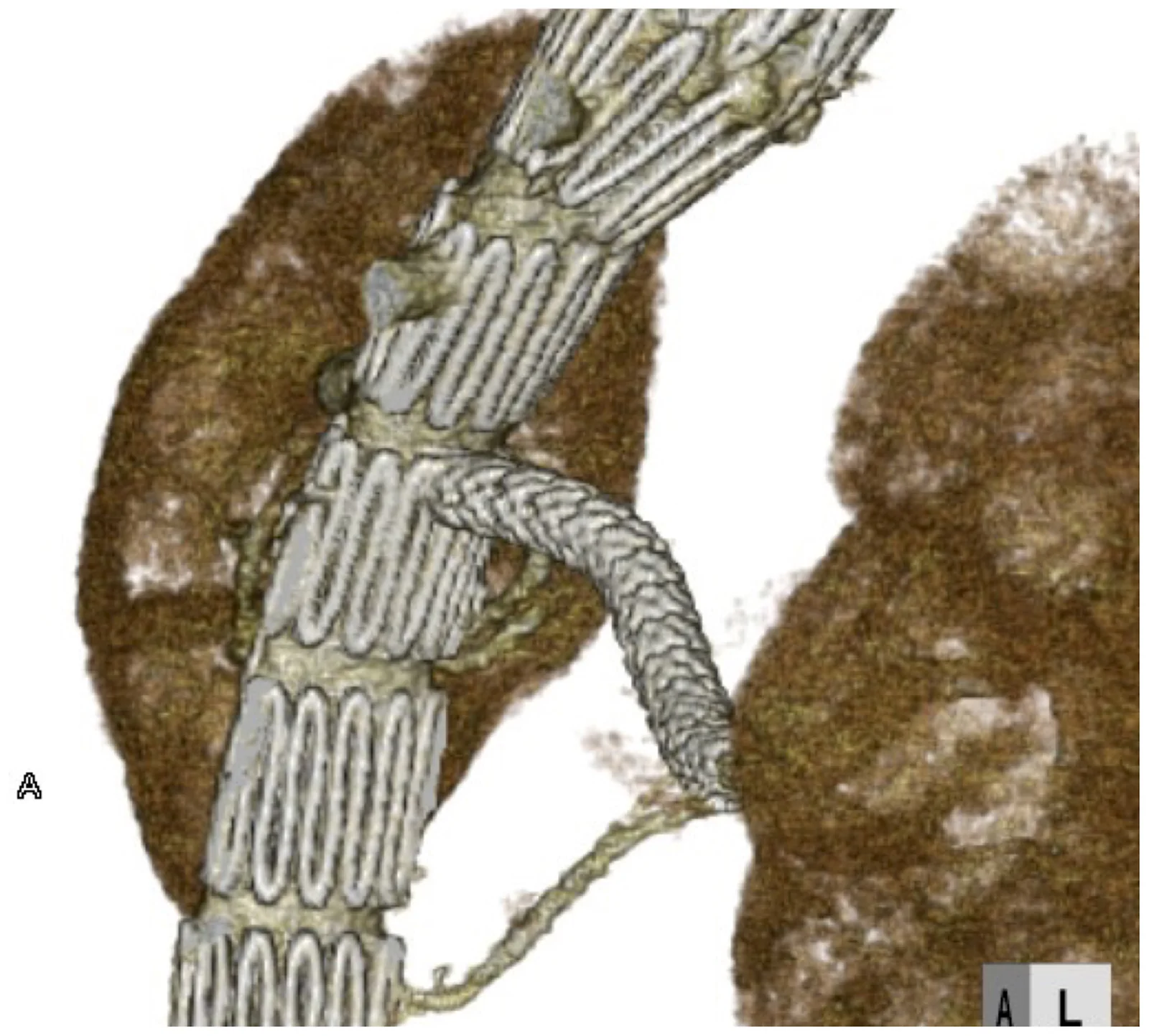
Postoperative three-dimensional volume-rendered computed tomography (CT) illustrating the spatial relationship among the provisional extension to induce complete attachment (PETTICOAT) bare-metal stent struts, the renal artery-associated re-entry tear, and the deployed VBX-covered stent. The VBX traverses the re-entry tear located near the base of a downward-facing triangular stent cell.

## Discussion

The PETTICOAT technique, combining TEVAR with distal bare-metal stents, improves early aortic remodeling and promotes false-lumen thrombosis.^1^^,^^2^ Despite these benefits, late aneurysmal degeneration remains problematic, with approximately 21% of patients requiring reintervention for distal aortic enlargement. Aortic growth predominantly occurs in the bare-stented abdominal aorta, with mean abdominal aortic volume increases of up to 31%.^3^ Persistent false-lumen perfusion through distal re-entry tears or visceral branch vessels often necessitates secondary intervention. However, the presence of a bare-metal stent can make endovascular reintervention after PETTICOAT technically challenging. In reports of fenestrated and branched endovascular aortic repair after PETTICOAT, the existing bare-metal stent has been shown to interfere with wire routing, catheter manipulation, and advancement of bridging stent delivery systems.^4^ Distal false-lumen perfusion is generally maintained by multiple distal re-entry tears or fenestrations. Although the exact number varies considerably among patients and according to the imaging criteria used, a CT-based study of type B aortic dissection reported a mean of 2.8 ± 2.11 intimomedial tears per patient.^5^ In our case, preoperative CT revealed a solitary re-entry tear adjacent to the left renal artery without any other identifiable source of persistent false-lumen perfusion, representing a relatively uncommon anatomical configuration that was amenable to treatment with a single covered stent. We therefore performed renal artery covered stent placement to achieve re-entry exclusion and preserve renal perfusion.

A VBX was selected instead of a self-expanding stent graft (VIABAHN; W. L. Gore & Associates) because of its smoother surface, greater rigidity, and superior deliverability, particularly if the guiding sheath could not be sufficiently advanced into the left renal artery. Device sizing was determined according to the diameter of the left renal artery rather than the entry diameter. Excessive oversizing within the renal artery could induce renal artery dissection, a difficult complication to salvage endovascularly. In contrast, undersizing relative to the entry diameter could be addressed with additional postdilation if necessary.

Preoperative CT evaluation of the spatial relationship between the re-entry hole and PETTICOAT stent struts is essential for procedural planning. Specifically, practitioners should assess the relationship of the entry hole to the apex or base of the triangular stent cell configuration before approach-route selection. If the entry hole is located near the apex, the guiding sheath may become caught on the stent strut, depending on the sheath orientation, potentially limiting device delivery or causing incomplete stent-graft expansion. In the present case, the re-entry hole was located near the base of a downward-facing triangular stent cell; therefore, a retrograde approach from the distal side was selected for easier access and fewer delivery constraints. Although slight proximal migration during deployment resulted in two stent rings entering the true lumen, protrusion of a single independent VBX stent ring may be preferable to prevent further device movement.

## Conclusions

Persistent false-lumen perfusion through a renal artery re-entry may contribute to late aneurysmal degeneration after PETTICOAT. As PETTICOAT usage increases, the technical challenges of visceral branch re-entry and secondary endovascular intervention should become increasingly important. Careful procedural planning and renal artery covered stent placement may offer a feasible strategy for branch-preserving reintervention.

## Funding

Not applicable.

## Disclosures

The authors declare that they have no competing interests.
